# Increased Isolation Frequency of Toxigenic *Vibrio cholerae* O1 from Environmental Monitoring Sites in Haiti

**DOI:** 10.1371/journal.pone.0124098

**Published:** 2015-04-08

**Authors:** Meer T. Alam, Thomas A. Weppelmann, Ira Longini, Valery Madsen Beau De Rochars, John Glenn Morris, Afsar Ali

**Affiliations:** 1 Department of Environmental and Global Health, College of Public Health and Health Professions, University of Florida, Gainesville, Florida, United States of America; 2 Emerging Pathogens Institute, University of Florida, Gainesville, Florida, United States of America; 3 Department of Biostatistics, Colleges of Medicine and Public Health and Health Professions, University of Florida, Gainesville, Florida, United States of America; 4 Department of Health Services Research Management and Policy, University of Florida, College of Public Health and Health Professions, Gainesville, Florida, United States of America; 5 Department of Medicine, College of Medicine, University of Florida, Gainesville, Florida, United States of America; Louisiana State University and A & M College, UNITED STATES

## Abstract

Since the identification of the first cholera case in 2010, the disease has spread in epidemic form throughout the island nation of Haiti; as of 2014, about 700,000 cholera cases have been reported, with over 8,000 deaths. While case numbers have declined, the more fundamental question of whether the causative bacterium, *Vibrio cholerae* has established an environmental reservoir in the surface waters of Haiti remains to be elucidated. In a previous study conducted between April 2012 and March 2013, we reported the isolation of toxigenic *V*. *cholerae* O1 from surface waters in the Ouest Department. After a second year of surveillance (April 2013 to March 2014) using identical methodology, we observed a more than five-fold increase in the number of water samples containing culturable *V*. *cholerae* O1 compared to the previous year (1.7% vs 8.6%), with double the number of sites having at least one positive sample (58% vs 20%). Both seasonal water temperatures and precipitation were significantly related to the frequency of isolation. Our data suggest that toxigenic *V*. *cholerae* O1 are becoming more common in surface waters in Haiti; while the basis for this increase is uncertain, our findings raise concerns that environmental reservoirs are being established.

## Introduction

Epidemic cholera was first identified in Haiti in October of 2010, with an altered toxigenic *V*. *cholerae* O1 El Tor biotype responsible for the sudden outbreak [1,2]. At the beginning of this study in April 2013, over 650,000 cholera cases and 8,000 deaths had been reported to the Haitian Ministry of Public Health and Population (MSPP) [3]. Although the number of reported cholera cases continues to decline, cholera remains an important cause of morbidity and mortality, with almost 50,000 cases reported in Haiti between April 2013 and March 2014 [4]. The government of Haiti, in collaboration with the Dominican Republic and the Pan American Health Organization (PAHO) has developed a plan for eradication of cholera from Haiti by 2022, which relies mainly on strategic immunization and improvements in sanitation [5]. A recent model reported by Bertuzzo et al [6], which did not incorporate the ability of toxigenic *V*. *cholerae* to establish environmental reservoirs, suggests that while eradication is possible, the likelihood of eradication will remain low without carefully targeted and effective interventions. However, if *V*. *cholerae* were able to establish environmental reservoirs as it has in other tropical countries [7,8], eradication of the disease from Haiti would become much less likely, if not impossible. To assess the possible role of surface water in cholera transmission, and to explore whether *V*. *cholerae* has established environmental reservoirs in Haiti, our research group at University of Florida has monitored 15 fixed environmental sites in the Ouest Department of Haiti on a monthly basis since April 2012. The observations made during the initial year of surveillance from April 2012 through March 2013 documented the isolation of toxigenic *V*. *cholerae* O1 from 3 of 179 (1.7%) water samples [9]. In the current report, the results of a second year of environmental surveillance performed during April 2013 to March 2014 are presented.

## Materials and Methods

### Water sampling sites

As a component of our ongoing surveillance for the isolation of *V*. *cholerae* strains in the Gressier/Leogane regions in Haiti, we selected 15 environmental sentinel sites as described previously [9]. The original 15 sites in the Leogane flood basin included locations along transects of the Momance and Gressier Rivers (4 up-river sites and one estuarine site at the mouth of each river), the Tapion River (4 river sites), and an independent estuarine site at Four-a-chaux. In addition to the original 15 sites, two sites were added that include a populated market in Gressier (R2S5) directly after Gressier (R2S4), and the Reserved (Res), a site downstream from Bellvue that receives water from the Taipon River and a nearby spring. Water samples were collected from the 17 environmental sites during the last week of every month from April 2013 to March 2014. Water sampling (Field work) was conducted with the knowledge of health authorities at the Haitian National Public Health Laboratories. All collection sites were at locations that were open to the general public (rivers and shoreline areas); specific permissions were not required for collection of water samples from these sites. Field studies did not involve collection of any animals, endangered or protective species.

### Isolation and characterization of *V*. *cholerae* from water samples

Water samples were collected from 17 environmental sites on a monthly basis from April 2013, through March 2014; a sterile Nalgene bottle (500 ml) was used to collect water from each site before transportation to the University of Florida field laboratory in Gressier for analysis. All samples were transported at ambient temperature and arrived within three hours of collection. Because of difficulty in accessing some sites at various time points due to seasonal fluctuations in water levels or road conditions, 197 water samples were available for analysis.

To isolate *V*. *cholerae* from collected water sample, a modified enrichment technique was used as described previously [9]. Briefly, three 1.5 ml aliquots from each water sample were enriched with equal volumes of 2X alkaline peptone water (APW) and incubated at 37°C for 6–8 h, 18–24 h at 37°C, and at 40°C for 6–8 h. Following enrichment, a sterile loop was used to streak cultures onto thiosulfate citrate bile salts sucrose (TCBS) agar (Becton, Dickinson and Company, NJ, USA) and the cultures were incubated at 37°C for 16–24 h. Six to eight yellow colonies from TCBS agar were transferred to L-agar plates and the plates were incubated at 37°C for 16–24 h. Colonies were screened for oxidase activity and oxidase-positive colonies were subjected to further screening using polyvalent *V*. *cholerae* O1-specific and O139 specific antisera (DENKA SEIKEN Co., Ltd., Tokyo, Japan) using slide agglutination assay.

### Genetic characterization

For further characterization, each isolate was examined using polymerase chain reaction (PCR) to amplify *ompW* and *toxR* using *V*. *cholerae* species specific primers as previously described [10]. Chromosomal DNA was extracted, using a GenElute Bacterial Genomic DNA kit (Sigma-Aldrich, St. Louis, MO), from each isolate exhibited positive PCR results for *ompW* and *toxR*. DNA was then used as a template for PCR amplification for key virulence genes, including *ctxA*, *ctxB*, *rstR*, *rstC*, and *tcpA* for both classical and El Tor genotypes as described previously [10,11]. Convergent PCR primers used in this analysis are listed in S1 Table. A standard membrane filtration technique was used to enumerate the concentrations (colony forming units per 100 ml) of fecal coliform bacteria in the water samples [12]. During the collection of water samples, water temperature of each site was also recorded.

### Antibiotic susceptibility test (AST)

Antimicrobial susceptibility test (AST) was performed for each isolate against a battery of antimicrobial agents using disc diffusion (Kirby-Bauer) assay and following the recommended guidelines by Clinical and Laboratory Standards Institute (CLSI, formally the National Committee for Clinical Laboratory Standards, NCLLS) as previously described [13]. Briefly, a small inoculum of each bacterial isolate was emulsified in 3 ml of sterile normal saline in 10 ml glass tube and the density of the bacterial suspension was compared with a barium chloride standard (0.5 Mcfarland). Subsequently, a sterile cotton swab stick was dipped into the standardized bacterial culture and the culture was evenly spread on the Mueller-Hinton plates and the plates were allowed to dry. Antibiotic discs (Oxoid, Basingstoke, England) with the following drug concentrations, including nalidixic acid (30 μg), tetracycline (30 μg), doxycycline (30 μg), amoxicillin (30 μg), ampicllin (30 μg), trimethroprim-sulfamethoxazole (cotrimoxazole) (25 μg), ciprofloxacin (5 μg), and chloramphenicol (30 μg) were placed on the plates. Discs were placed at least 15 mm apart and from the edge of the plates to prevent the overlapping of zones of inhibition. Plates were incubated at 37°C for 18–24 h, and the diameters of zones of inhibition of each antibiotic were compared with the recorded diameters of the control organism *E*. *coli* ATCC 25922 to determine susceptibility, intermediate or resistance pattern of the *V*. *cholerae* isolate to that antibiotic.

### Statistical analysis

The water temperatures from all of the collection sites were averaged to obtain monthly estimates of the water temperature in degrees Celsius (°C). Daily estimates of rainfall in millimeters per day collected by the Tropical Rainfall Measuring Mission (TRMM) satellite were obtained from the National Aeronautics and Space Administration (NASA) for the area directly proximal to the environmental sampling sites (18.2–18.5°N, 17.1–17.4°W) and aggregated into weekly accumulated precipitation (mm/week) [14]. The association between the isolation of toxigenic *V*. *cholerae* O1 from individual water samples (presence/absence) and fecal coliform concentrations was assessed using conditional logistic regression with the site of collection (n = 17) as the grouping variable. A binomial regression model with time-lagged covariates was used to determine the association between the frequency of isolation of toxigenic *V*. *cholerae* O1 from all 17 collection sites and the monthly average water temperature and the weekly accumulated precipitation. The raw data used for the statistical analysis is available in S1 Dataset.

## Results and Discussion

Between April 2013 and March 2014, toxigenic *V*. *cholerae* O1was isolated from 17 (8.6%) of 197 surface water samples and from 10 (59%) of the 17 sample collection sites. The date of isolation, unique strain identification number associated with the isolate, the abbreviated site name, the full name of the collection site, and the concentration of fecal coliform bacteria isolated from the same water sample are presented in Table 1. Unlike the *V*. *cholerae* O1 strains isolated from April 2012 to March 2013, where four of the seven environmental *V*. *cholerae* O1 isolates lacked the entire cholera toxin phage (CTXɸ) and had classical variants of the toxin co-regulated pilus (*tcpA*), all 17 toxigenic *V*. *cholerae* O1 strains isolated between April 2013 and March 2014 had the altered El Tor biotype that has come to be associated with the Haitian cholera epidemic. This biotype includes a combination of El Tor biotype specific *tcpA* ^ET^, *rstR* ^ET^, and *ctxA* genes and a classical variant of the beta subunit of cholera toxin gene (*ctxB* ^CL^) (Table 2). Interestingly and in contrast to our first year survey [9], we observed that 12 (70.6%) of 17 isolates exhibited *rstC* ^ET^ genotype (Table 2).

**Table 1:** Summary information of environmental *V*. *cholerae* O1 isolates.

Months^*a*^	Strain	Site code	Site name	Fecal Coliforms^*b*^
				(CFU/ 100 ml)
April	-	-	-	-
May	-	-	-	-
June	env898	R1S1	Jean-Jean	80
	env901	R1S3	Bongnotte	200
	env894	R1S4	Brach	210
July	env949	R1S1	Jean-Jean	560
	env955	R1S4	Brach	560
	env961	BEL	Bellvue	710
August	env1054	R1S4	Brach	1310
September	env1183	R2S1	Upper Colin	560
	env1180	R2S2	Colin	1700
	env1177	R2S3	Sousfort	800
	env1112	CVB	Christianville bridge	890
October	env1239	R1S1	Jean-Jean	1100
	env1222	R1S3	Bongnotte	710
	env1218	R1S4	Brach	730
	env1231	RES	Reserved	690
November	env1320	CVS	Christianville spring	100
	env1321	RES	Reserved	800
December	-	-	-	-
January	-	-	-	-
February	-	-	-	-
March	-	-	-	-

^*a*^Monthly sampling occurred at the 17 fixed sites between April 2013 and March 2014

^*b*^Fecal coliform bacteria were enumerated using a standard membrane filtration technique

**Table 2:** PCR analysis of key genes of toxigenic *V*. *cholerae* O1 strains isolated from environmental samples in Haiti.

	Key genes amplified by PCR^*a*^										MAMA-PCR^*b*^	
Strains^*c*^	*ompW*	*toxR*	*tcpA* ^CL^	*tcpA* ^ET^	*ctxA*	*ctxB*	*rstR* ^ET^	*rstR* ^CL^	*rstC* ^ET^	*rstC* ^CL^	*ctxB* ^CL^	*ctxB* ^ET^
env894	+	+	-	+	+	+	+	-	+	-	+	-
env898	+	+	-	+	+	+	+	-	+	-	+	-
env901	+	+	-	+	+	+	+	-	+	-	+	-
env949	+	+	-	+	+	+	+	-	+	-	+	-
env955	+	+	-	+	+	+	+	-	+	-	+	-
env961	+	+	-	+	+	+	+	-	+	-	+	-
env1054	+	+	-	+	+	+	+	-	+	-	+	-
env1112	+	+	-	+	+	+	+	-	-	-	+	-
env1177	+	+	-	+	+	+	+	-	-	-	+	-
env1180	+	+	-	+	+	+	+	-	-	-	+	-
env1183	+	+	-	+	+	+	+	-	+	-	+	-
env1218	+	+	-	+	+	+	+	-	+	-	+	-
env1222	+	+	-	+	+	+	+	-	+	-	+	-
env1231	+	+	-	+	+	+	+	-	+	-	+	-
env1239	+	+	-	+	+	+	+	-	-	-	+	-
env1320	+	+	-	+	+	+	+	-	-	-	+	-
env1321	+	+	-	+	+	+	+	-	+	-	+	-

^*a*^Genetic characterization was performed by PCR using convergent primer sets specific for both El Tor and classical *V*. *cholerae* O1 strains.

^*b*^All isolates are *V*. *cholerae* O1 biotype El-Tor strains carrying classical *ctxB* gene as determined by MAMA-PCR.

^*c*^Twelve (70.6%) of the 17 isolates exhibited the presence of *rstC* ^ET^ genotype

As described in Methods section, we used diverse APW enrichment techniques in order to enhance increased *V*. *cholerae* isolation from water samples collected in Haiti. Data, presented in Table 3, demonstrated that if we were to use only conventional APW enrichment technique [15], we would have missed 6 (35%) of the 17 toxigenic *V*. *cholerae* strains reported in this investigation. Given the difficulty of the isolation of culturable *V*. *cholerae* from aquatic reservoirs [16], our modified APW enrichment approach played a crucial role for the increased isolation of toxigenic *V*. *cholerae* in Haiti.

**Table 3:** Effect of diverse enrichment conditions for the isolation of culturable *V*. *cholerae* O1 strains from aquatic reservoirs in Haiti.

Cultures positive V. cholerae O1 strains isolated after APW enrichment at diverse incubation conditions			
Strain ID			
	37°C (6–8 h)	37°C (18–24 h)	40°C (6–8 h)
env894	**+**	**-**	**+**
env898	**+**	**-**	**+**
env901*	-	-	+
env949*	-	-	+
env955	+	-	+
env961	+	-	-
env1054	+	-	+
env1112**	-	+	+
env1177**	-	+	+
env1180	+	+	+
env1183	+	+	+
env1218	+	+	-
env1222***	-	+	-
env1231	+	-	-
env1239	+	-	+
env1320**	-	+	+
env1321	+	+	+

*Isolates exhibited growth only in APW cultures incubated at 40°C for 6–8 h

**Isolates exhibited growth in APW cultures incubated both at 40°C for 6–8 h and at 37°C for 18–24h

***Isolates exhibited growth only in APW cultures incubated at 37°C for 18–24 h

In order to determine the susceptibility of *V*. *cholerae* isolates to antibiotics, each *V*. *cholerae* strain was subjected to antibiotic susceptibility test to a battery of antibiotics (Table 4). All 17 isolates exhibited identical response to each antibiotic examined. Environmental Haitian *V*. *cholerae* O1 strains were resistant to nalidixic acid and SXT (cotrimazole) and showed reduced susceptibility to ampicillin and chloramphenicol. However, they were sensitive to doxycycline, tetracycline and ciprofloxacin. Our data are similar to results presented in a previous study where Haitian clinical strains were subjected to antibiotic susceptibility assay [17].

**Table 4:** Antibiotic susceptibility test result.

Antibiotics	Sensitive (S)	Resistant (R)	Intermediate (I)
Nalidixic acid		R	
Ampicillin			I
Chloramphenicol			I
Doxycycline	S		
SXT (cotrimazole)		R	
Tetracycline	S		
Ciprofloxacin	S		

**Fig 1:**
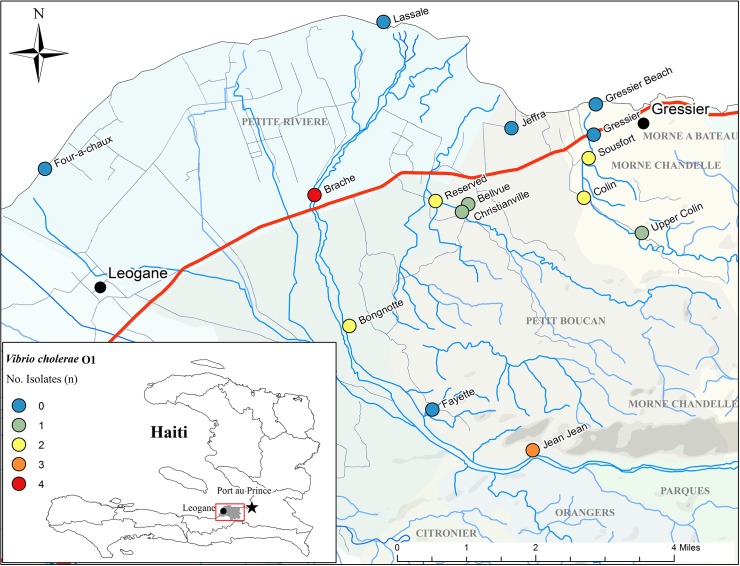
Map displaying location of environmental sampling sites from the Leogane flood basin (gray shaded region, inset) in the Ouest Department of Haiti and the source of rainfall measurements bounded by the region 18.2–18.5°N, 17.1–17.4°W (red square inset). The environmental samples were collected between the months of April, 2013 and March, 2014. The number of V. cholerae O1 isolates obtained from each sampling site is differentiated by color.

In contrast to the previous year, where *V*. *cholerae* O1 was exclusively isolated in estuarine sites at the mouths of the Taipon and Momance Rivers (Lassale, Jeffra, and Gressier Beach); in the current study *V*. *cholerae* O1 was only isolated from upstream collection sites, which included a transect of the Momance River approaching Mount Chandelle (Jean-Jean). The geographic locations of the fixed collection sites where toxigenic *V*. *cholerae* O1 were isolated are presented with respect to isolation frequency in Fig 1. The likelihood of isolating *V*. *cholerae* from the environment was not increased with higher concentration of fecal coliform bacteria in the water samples (*P* > 0.5).

**Fig 2:**
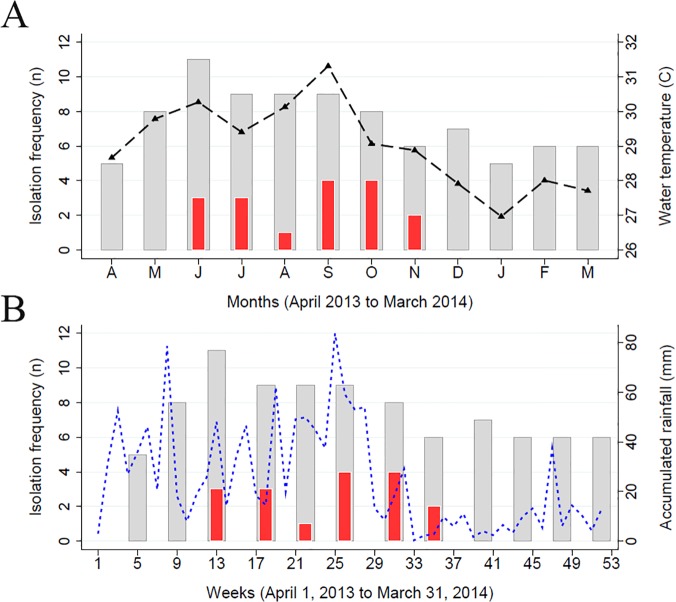
The isolation frequency of both toxigenic *V*. *cholerae* O1 (red bars) and non-toxigenic *V*. *cholerae* Non-O1/O139 (gray bars) appear with respect to time overlaid with average monthly water temperature in degrees Celsius (panel A) and accumulated weekly rainfall in millimeters (panel B).

The isolation frequency of *V*. *cholerae* O1 and non-O1/O139 from all 17 collection sites is presented by month along with the average water temperature in Fig 2A. In contrast to the year-round isolation of non-toxigenic *V*. *cholerae* non-O1/O139 from the environment, the isolation of *V*. *cholerae* O1 only occurred during the early summer and late fall (June to November), corresponding with increases in water temperatures. The average water temperature was highly correlated with the isolation frequency of *V*. *cholerae* O1 both during the same month (*P* = 0.002) and the following month (*P* < 0.001). For each additional one degree Celsius increase (°C) in the average water temperature, the frequency of isolation was almost twice as high (RR = 1.87; 95% CI: 1.27, 2.76) during the same month and 2.2 times as high (RR = 2.23; 95% CI: 1.46, 3.39) the following month. The isolation frequency of *V*. *cholerae* O1 and Non-O1/O139 from all 17 collection sites is presented by week along with accumulated rainfall in Fig 2B. As shown, both toxigenic *V*. *cholerae* O1 and *V*. *cholerae* non-O1/O139 were isolated more frequently during the rainy season (approximately from mid-April to mid-September), however toxigenic *V*. *cholerae* O1 was isolated exclusively after the onset of the rainy season until November when the precipitation decreased. The isolation frequency of *V*. *cholerae* O1 from the environment increased significantly four to five weeks after increases in accumulated rainfall (*P* = 0.001, 0.002) and also showed a smaller, less significant increase during the same week (*P* = 0.036). For each additional 10 mm of accumulated rainfall, there was a 27% increase in isolation frequency during the same week (RR = 1.27, 95% CI: 1.02, 1.58), a 48% increase four weeks later (RR = 1.48, 95% CI: 1.17, 1.87), and a 34% increase five weeks later (RR = 1.12, 1.60).

Despite a decrease in the number of cholera cases reported in the two nearest cities (Gressier and Leogane) from approximately 3,000 cases during 2012/13 to 965 cases from 2013/14 [personal communication, MSPP], the isolation rate of toxigenic *V*. *cholerae* O1 from the environment has increased more than five-fold from 1.7% (3/179) to 8.6% (17/179) during the same period of time [4]. The geographic area where toxigenic *V*. *cholerae* O1 strains were isolated also appears to have expanded to include over half of the collection sites, with isolations taking place further up-river. Furthermore, the temporal pattern of isolation has begun to demonstrate seasonal correlations with increases in precipitation and water temperature. This corresponds with observations from countries with endemic *V*. *cholerae* O1, where seasonal increases in water temperature or the onset of the rainy season serve as triggers for the proliferation of *V*. *cholerae* O1 in the environment that precede seasonal cholera epidemics [15,18–19]. The additional inclusion of fecal coliform bacteria has also indicated that though fecal coliforms were present in all surface water samples, there was not an increased likelihood of isolating toxigenic *V*. *cholerae* at higher fecal coliform concentrations, and that *V*. *cholerae* was isolated in water samples with relatively low concentrations of fecal coliforms (< 1 CFU/ml). Regardless of whether toxigenic *V*. *cholerae* O1 have established true environmental reservoirs or the surface water was contaminated by those infected with cholera, the presence of toxigenic *V*. *cholerae* O1 in the surface water represents a potential ongoing source of exposure.

## Supporting Information

S1 Table S1 Table contains a list of the oligonucleotide primers used in polymerase chain reactions (PCR) for detection and characterization of toxigenic *Vibrio cholerae* O1. (DOCX)

S1 Dataset S1 Dataset contains a the sample collection date, epidemiological week from the beginning of the sampling period, the number of weekly reported cases, the frequency of *V*. *cholerae* O1 and non O1 isolations per week, the average monthly water temperature (°C), and the accumulated weekly precipitation (mm/week). (XLSX)
