# Supplementary material for: Increased Isolation Frequency of Toxigenic Vibrio cholerae O1 from Environmental Monitoring Sites in Haiti
Source: PLoS One. 2015 Apr 8;10(4):e0124098. doi: 10.1371/journal.pone.0124098 (PMC4390201; doi:10.1371/journal.pone.0124098)
Supplement: S1 Table — (DOCX) [file pone.0124098.s001.docx]

| Primer Name | Direction | Target gene | Sequence (5’ to 3’) |
| --- | --- | --- | --- |
| aa048 | Forward | *ompW* | CACCAAGAAGGTGACTTTATTGTG |
| aa049 | Reverse |  | GGTTTGTCGAATTAGCTTCACC |
| aa072 | Forward | *toxR* | TGCCTTCATCAGCCACTGTAGT |
| aa073 | Reverse |  | AGCAGTCGATTCCCCAAGTTT |
| aa078 | Forward | *ctxA* | CTCAGACGGGATTTGTTAGGCACG |
| aa079 | Reverse |  | TCTATCTCTGTAGCCCCTATTACG |
| aa044 | Forward | *ctxB* | AAAATTCCTTGACGAATACC |
| aa045 | Reverse |  | TTGCTTCTCATCATCGAACC |
| aa100 | Forward | *tcpA* | AAAACCGGTCAAGAGGG |
| aa101 | Reverse | *tcpA*^ET^ | CAAAAGCTACTGTGAATGG |
| aa102 | Reverse | *tcpA*^CL^ | CAAATGCAACGCCGAATGG |
| aa145 | Forward | *ctxB* | ACTATCTTCAGCATATGCACATGG |
| aa146 | Reverse | *ctxB*^ET^ | CCTGGTACTTCTACTTGAAACG |
| aa147 | Reverse | *ctxB*^CL^ | CCTGGTACTTCTACTTGAAACA |
| aa196 | Forward | *rstR*^CL^ | CTTCTCATCAGCAAAGCCTCCATC |
| aa197 | Forward | *rstR*^ET^ | GCACCATGATTTAAGATGCTC |
| aa200 | Reverse | *rstC* | GCTCAGTCAATGCCTTGAGTTG |
| aa201 | Reverse | *rstA* | GCATAAGGAACCGACCAAGCAAGAT |

Table S1. List of PCR primers used in this study
